# Electrospun Nanofiber Materials for Photothermal Interfacial Evaporation

**DOI:** 10.3390/ma16165676

**Published:** 2023-08-18

**Authors:** Dianming Li, Yingying Cheng, Yanxia Luo, Yuqin Teng, Yanhua Liu, Libang Feng, Nü Wang, Yong Zhao

**Affiliations:** 1 School of Materials Science and Engineering, Lanzhou Jiaotong University, Lanzhou 730070, China; lidm@lzjtu.edu.cn (D.L.); yx0912in@163.com (Y.L.); happyliuyanhua@126.com (Y.L.); 2 Key Laboratory of Bioinspired Smart Interfacial Science and Technology of Ministry of Education, School of Chemistry, Beihang University, Beijing 100191, China

**Keywords:** electrospun, nanofiber, hierarchical structure, photothermal interfacial evaporation

## Abstract

Photothermal interfacial evaporation with low cost and environmental friendliness has attracted much attention. However, there are still many problems with this technology, such as heat loss and salt accumulation. Due to their different structures and adjustable chemical composition, electrospun nanofiber materials generally exhibit some unique properties that provide new approaches to address the aforementioned issues. In this review, the rational design principles for improving the total efficiency of solar evaporation are described for thermal/water management systems and salt-resistance strategies. And we review the state-of-the-art advancements in photothermal evaporation based on nanofiber materials and discuss their derivative applications in desalination, water purification, and power generation. Finally, we highlight key challenges and opportunities in both fundamental research and practical applications to inform further developments in the field of interfacial evaporation.

## 1. Introduction

The demand for resources is rising as society and the economy develop [1]. Freshwater is a resource on which human beings depend and is indispensable for our survival. The existing freshwater accounts for only 2.5% of the global water resources [2], while the portion of rivers, lakes, and groundwater that can actually be used accounts for only 0.325% of the total amount of water on Earth. According to the UN data, 1.1 billion people globally endure water scarcity, and 2.6 billion people lack access to basic sanitation [3]. Given the increasing depletion of non-renewable resources, it is very necessary to produce water in a sustainable manner in order to meet current resource demands.

Traditional desalination technologies [4,5,6,7,8,9] mainly include nanofiltration, reverse osmosis, multi-effect distillation, and multistage flash evaporation, all of which have shortcomings such as high desalination costs, secondary pollution, and high energy consumption [10]. Photothermal interfacial evaporation (PIE) technology has been proposed as a viable solution to the freshwater crisis in recent years [11,12]. The PIE system can confine the heat to the water–air interface while inhibiting heat transfer to the bulk water [13], demonstrating a significant application potential. However, conventional PIE suffers from heat loss and poor salt resistance, resulting in a low photothermal evaporation efficiency. Photothermal evaporation films have attracted much attention as critical parts of interfacial evaporation devices because of their easy fabrication, rich variety, and scalability. Methods for phase transition, sol-gel, and freeze-drying can be used to prepare conventional membrane materials, which are time-consuming and complex [14]. In contrast, electrospun nanofibers are flexible, lightweight, and durable [15], facilitating large-scale applications. The entangled porosity structure allows for multiple light reflections, which improves sunlight absorption and also creates a path for vapor to escape. As a result, the application of electrospun nanofiber materials in PIE has great prospects [16,17,18].

**Figure 1:**
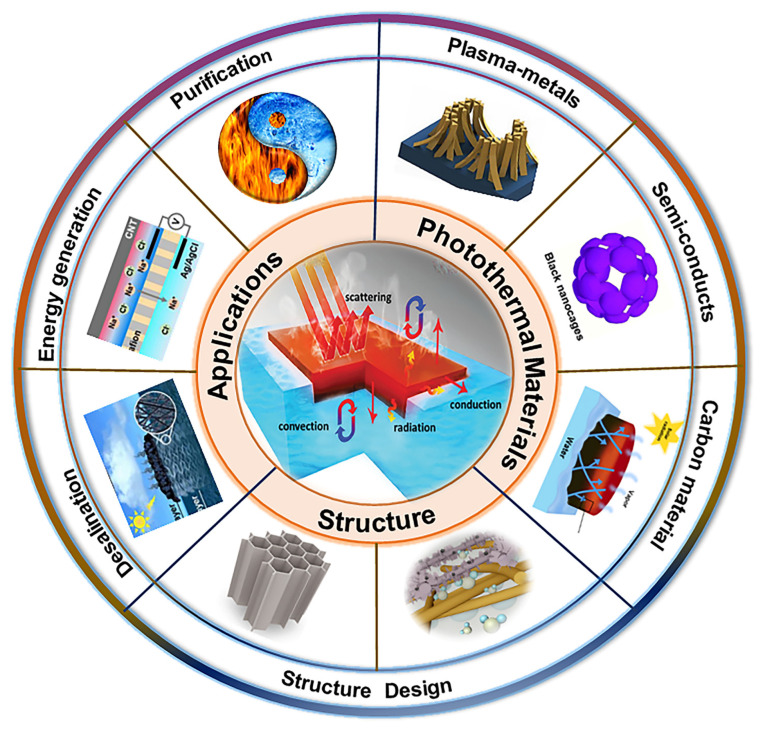
Schematic illustration of the PIE. An overview of PIE, including: (1) the materials about plasma metals, semiconductors, and carbon materials; (2) the structural design of photothermal evaporators and the impact on thermal and water management; (3) the applications in desalination and accompanying desalination processes for energy conversion, water purification, and photocatalysis.

In this review, the development of research on photothermal conversion materials, PIE systems, and diverse photothermal conversion methods are presented methodically. The design criteria for efficient PIE materials are discussed in terms of photothermal conversion, thermal management, water management, and salt-resistance performance. In addition, the PIE systems based on two-dimensional (2D) membrane materials and three-dimensional (3D) aerogel materials are systematically reviewed. Notably, we discuss the structural design of single fibers at the nanoscale and their application in PIE in the context of our group’s results in the field of multilevel structured fibers. Finally, derivative applications (energy conversion, photothermal catalysis, and wastewater treatment) of evaporation systems based on electrospun fibers accompanying the PIE process are described (Figure 1). The goal of this review is to provide direction for the design of PIE devices by effectively understanding the photothermal conversion and thermal mass transport mechanisms of multi-scale structured fiber materials.

## 2. Overview of Electrospun Nanofibers

**Figure 2:**
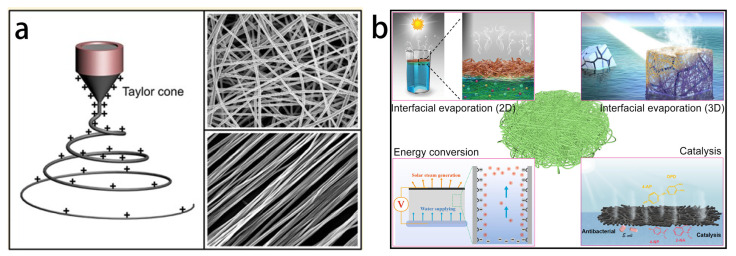
(**a**) Main components of a typical electrospinning device: spinneret, high-voltage power supply, and grounded conductive collector. SEM images of the electric field around the Taylor cone and its typical electrospun fibers. (**b**) Interfacial evaporation and derivative applications (energy conversion and catalysis) based on electrospun fiber materials. (**a**) Reproduced from Ref. [41].

Electrospinning [19,20,21] is a simple and versatile method for preparing fibrous materials. The basic principle is to stretch or melt a polymer solution by spraying it under the force of a high-voltage electric field and obtain ultrafine fibers using solvent evaporation or melt curing [22]. Due to the benefits of high porosity and adjustable structure, electrospinning has become a crucial technology to produce nanofibers [23]. The electrospinning device is made up of three basic parts: a grounded reception device, a syringe with polymer solutions, and a high-voltage power supply that generates a high-voltage electrostatic field force, as shown in Figure 2a. Currently, organic nanofibers, organic/inorganic hybrid composite nanofibers, inorganic nanofibers, and carbon nanofibers [24,25] are fabricated with electrospinning technology. These materials have been used extensively in the fields of filtration materials [26], energy materials [27], biomedicine [28], sensors [29], and photocatalysis [30].

In contrast to other innovative production methods for membranes, electrospinning has many advantages. First, electrospinning can convert many soluble polymers into fibrous films [31]. Second, by adjusting the solution concentration, voltage, distance, and other variables, the electrospinning can change the structure and shape of the fiber membrane [32]. This technique makes it simple to change variables like the membrane porosity and fiber diameter [33]. Additionally, using rational design of the spinneret, it is possible to generate multistage structured fibers with features including core–shell, spiral, tubular, beaded, and hollow structures (Figure 2b). Electrospun fiber membranes can also be further functionalized [34,35,36,37] by combining them with plasma treatment, atomic layer deposition, hydrothermal treatment, chemical vapor deposition, and low-surface-energy modification, considerably expanding the spectrum of applications for fiber materials [38,39,40,41,42].

## 3. Interface Evaporation Design Principles

The design of the interfacial evaporation system is particularly crucial to improving the photothermal conversion efficiency [43,44]. The construction of the interfacial evaporator mainly includes the selection of photothermal materials, optimization of water/heat management, and design of resistance to salt accumulation [45]. The ratio of energy required for steam generation to solar energy absorption can be calculated to obtain the photothermal conversion efficiency, as illustrated in the equation:

(1) $$\eta =\dot{m}{\;h}_{\mathrm{lv}}/P_{\mathrm{in}}$$

where $\dot{m}$ is the mass flow rate of steam, $h_{\mathrm{lv}}$ is the gas-liquid enthalpy change of water, and $P_{\mathrm{in}}$ is the energy of incident light.

Therefore, in order to achieve efficient evaporation, the PIE should meet three requirements: (1) efficient solar energy absorption; (2) superior thermal and water management; (3) durable salt resistance.

### 3.1. Highly Efficient Solar Energy Absorber

**Figure 3:**
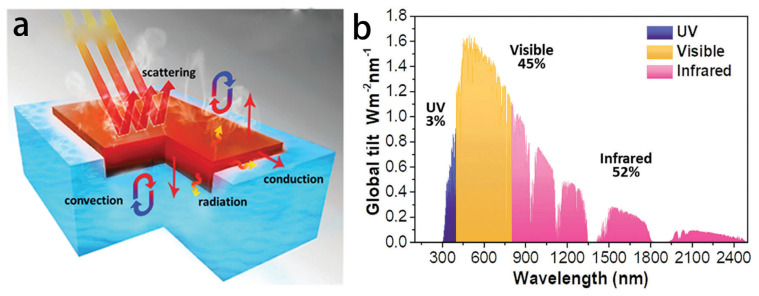
(**a**) Schematic diagram of the PIE system. (**b**) Solar spectrum irradiance (AM1.5). (**a**,**b**) Reproduced from Ref. [49].

Through photovoltaic [46], photochemical, and photothermal [15] processes, solar energy can be transformed into other forms of energy. A direct conversion procedure with a particularly high conversion efficiency among these technologies is photothermal technology. The photothermal effect is the process whereby light stimulation produces some or all of the thermal energy [47]. A PIE system typically consists of the following factors: a solar absorber layer, thermal insulation layer, water supply channel, and vapor escape channel [48]. The process of PIE typically involves the following steps: (1) incident light is absorbed and converted into heat; (2) liquid water percolates into the surface layer through designated channels under capillary forces; (3) heat from the sun evaporates the surface water and creates water vapor. Due to heat conduction and heat radiation, heat loss is unavoidable throughout these processes, which lowers the photothermal evaporation efficiency below 100% (Figure 3a). The ability of the material to capture light is a crucial element in photothermal conversion. The scope of absorption across the solar spectrum and the strength of absorption at each wavelength are two components of this. Figure 3b illustrates the power distribution of solar energy incident on the Earth’s surface for the solar spectrum (AM 1.5) [13]. Interfacial evaporation can achieve the best results when the photothermal material has a high absorption and a small transmittance and reflectance in the spectrum range.

Due to their excellent photothermal properties, noble metal materials [50], semiconductor materials [51], and carbon-based materials [52] are currently the most representative photothermal conversion materials. We classify these materials into three groups based on the mechanism of the electromagnetic radiation–matter interaction, namely, plasma local heating of metals [53], nonradiative relaxation of semiconductors [54], and thermal vibrations of molecules [55]. The absorption of solar energy can be increased by modifying the composition and surface design, while reducing the thermal radiation and optical reflection.

#### 3.1.1. Nanometallic Materials

Some metal nanoparticles exhibit bright colors due to the disappearance of wavelengths of light caused by the local surface plasmon resonance effect of the metal [50]. When the intrinsic frequency of the electrons on the metal surface and the frequency of the light coincide, plasmon resonance happens. Heat is created through the Joule mechanism as a result of the excited hot electrons oscillating in synchrony with the external electromagnetic field. The hot carriers rapidly transfer energy, heating the plasmon resonance through electron–electron scattering.

**Figure 4:**
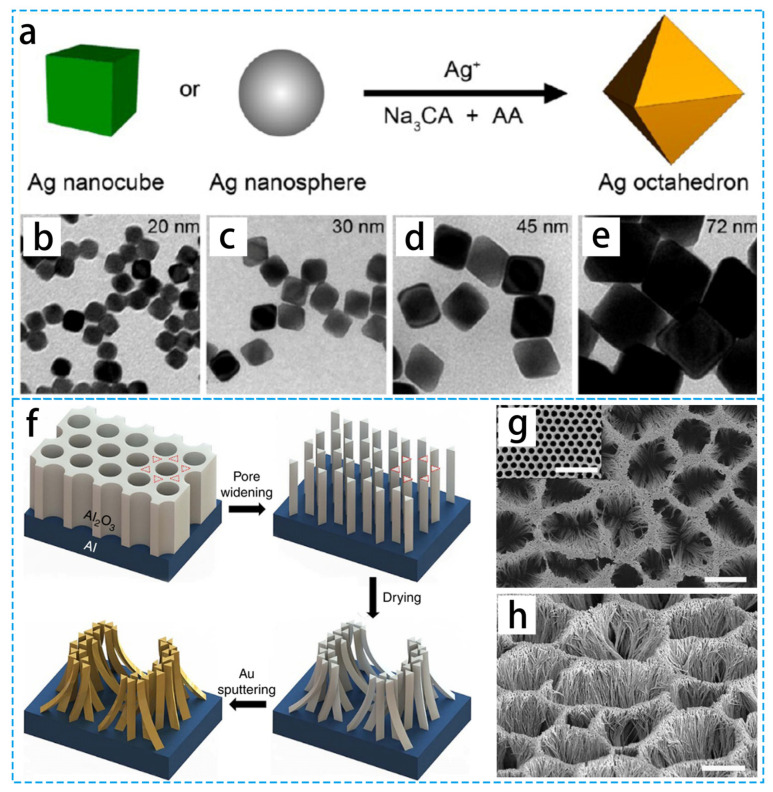
(**a**) Schematic diagram of single-crystal cubic silver seeds towards octahedra under standard conditions. (**b**–**e**) TEMs of octahedral silver with different edge lengths. (**f**) Schematic diagram and structure of the black gold film. (**g**,**h**) Top view of SEM image of black gold film (52° tilt) and ordered hexagonal array of AAO templates (inset **g**). Scale bars, 2 μm (**g**,**h**) and 500 nm (insert), respectively. (**a**–**e**) Reproduced from Ref. [56]; (**f**–**h**) Reproduced from Ref. [58].

Many researchers have been devoted to exploring the photothermal evaporation properties of plasma nanoparticles. As in Figure 4a–e, Xia’s group [56] introduced a method that can precisely control the size to synthesize silver octahedra. Gold nanocages with octahedral shapes and tunable optical properties were prepared using the obtained silver octahedra as sacrificial templates. Similarly, the photothermal nanofiber composite membrane for ultraviolet light-driven membrane distillation (MD) was demonstrated by Wang’s group [57]. In this, the photothermal nanoparticles, silver nanoparticles, were embedded in a porous membrane without the use of any other heating method, and the photothermal conversion was carried out at the membrane surface/water interface using light from a UV LED lamp, providing an efficient heating method. Except for noble metals, Figure 4f–h shows that the distinctive black gold loaded on anodic aluminum oxide (AAO) membranes exhibits ultra-broadband light absorption [58], leading to a high vapor-generation efficiency that absorbs a wide range of the solar spectrum (96%). Due to their excellent electromagnetic radiation absorption, metallic substances have been used extensively as PIE materials.

#### 3.1.2. Semiconductors

**Figure 5:**
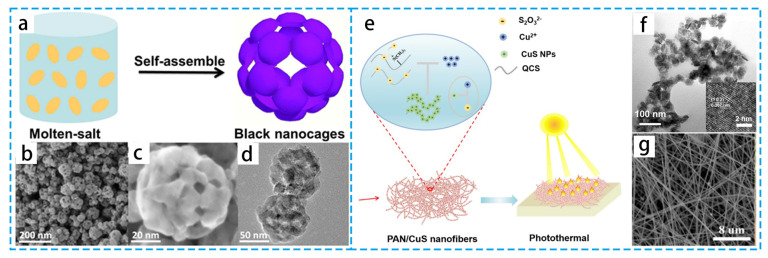
(**a**) Schematic diagram of black titanium dioxide nanospheres. (**b**,**c**) Characteristic SEM and (**d**) TEM images of the black titania nanocages. (**e**) The preparation of PAN/CuS fibrous membranes. (**f**) TEM image of copper sulfide (CuS) @quaternized chitosan NPs (CuS@QCS NPs). (**g**) SEM image of PAN/CuS nanofibers. (**a**–**d**) Reproduced from Ref. [54]; (**e**–**g**) Reproduced from Ref. [59].

Due to the tunable absorption spectra and high near-infrared extinction coefficients, many metal oxide and sulfur compound semiconductor photothermal nanomaterials show promising applications. Semiconductor materials can excite electrons to form electron–hole pairs when the energy of the incident photon is greater than or equal to the energy of the band gap. Following their return to the band-gap edge, the excited electrons subsequently release the remaining energy as thermal energy. The light-absorption ability of semiconductor materials is related to the free carriers. As shown in Figure 5a–d, instead of evenly heating the majority of the water, the self-floating black titanium dioxide nanocage covering concentrates the temperature increases at the water–air interface [54]. Based on the semiconductor effect, polyacrylonitrile (PAN)/CuS nanofiber films have good photothermal conversion properties in addition to a strong heating-cycle stability and high tensile strength [59] (Figure 5e–g). As efficient photothermal evaporation or catalytic materials, other semiconductors (SiO_2_ [60,61,62,63], Co_3_O_4_ [64], ZnO [65]) have also been reported.

#### 3.1.3. Carbon Materials

Materials based on carbon and various polymers exhibit high absorption over a broad wavelength range when compared to metallic and semiconductor materials. Due to the lower energy of π bonds compared to σ bonds, the energy gap between π and π* orbitals is significantly smaller and can be activated by very low-energy photons. The energy difference between the highest occupied molecular orbital and the lowest unoccupied molecular orbital becomes smaller as the number of conjugates rises. This eventually results in absorption over a broad wavelength range in carbon materials, which is then converted into thermal energy when the excited electrons return to the ground state.

**Figure 6:**
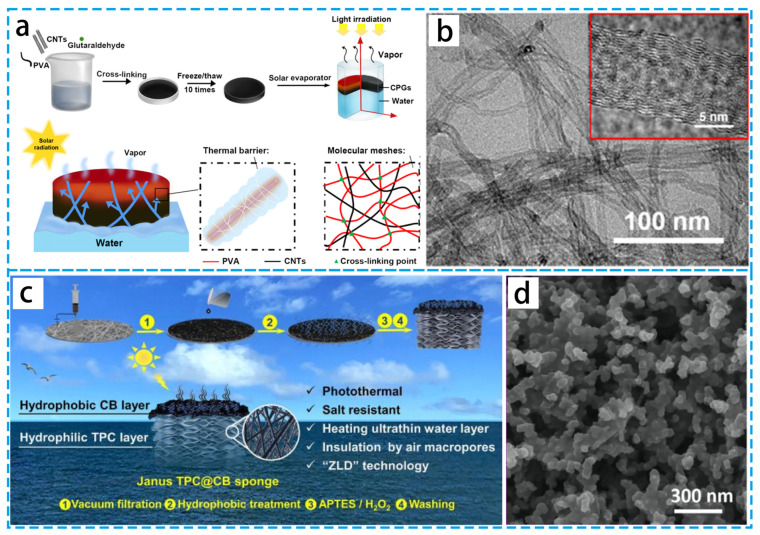
(**a**) Schematic diagram of the fabrication of 3D cross-linked CPG using a cyclic freeze-casting process for solar vapor generation based on efficient thermal localization. (**b**) TEM and HRTEM images of pure carbon nanotubes. (**c**) Schematic diagram of Janus TPC@CB sponge preparation process and its structure. (**d**) SEM image of TPC@CB sponge. (**a**,**b**) Reproduced from Ref. [66]; (**c**,**d**) Reproduced from Ref. [67].

Due to their broad range of competitive advantages, including thermal stability and environmental friendliness, carbon materials have emerged as the most promising photothermal materials. As shown in Figure 6a,b, hybrid gels (CPG) were prepared by freezing and thawing a mixture of CNTs and PVA [66]. Due to the porous PVA network’s superhydrophilic properties and the capillary effect, the multilayer PVA gel can effectively transfer water from internal capillary channels to molecular meshes. In addition, reduce heat loss and achieve thermal energy confinement to the water clusters by acting as an energy-absorbing medium with PVA molecular chains wrapped on the CNTs. As shown in Figure 6c,d, Jiao’s group [67] designed a self-floating Janus sponge made of a hydrophilic thermoplastic polyurethane–carbon black (TPC@CB) nanofiber substrate and a hydrophobic CB coating. The Janus TPC@CB sponge has a unique trilaminar functional structure: a superhydrophobic solar-absorbing coating at the top, an ultra-thin thermal localization layer, and a honeycomb insulation layer in the lower layer, which has a higher evaporation efficiency. Additionally, the treatment of saline wastewater using this Janus TPC@CB sponge as a PIE medium resulted in zero liquid discharge.

A self-contained solar energy converter was created by Chen’s group [52] using only cross-linked honeycomb graphene. This foam material can function as a PIE converter to distill water with a very high efficiency. Wang’s group [55] prepared a novel efficient solar vapor-generating light receiver consisting of reduced graphene oxide (rGO), polyurethane (PU), and a covalently cross-linked reduced graphene oxide nanosheet PU matrix with an outstanding stability and broad optical absorption. The rGO/PU foam has outstanding mechanical and chemical stability and PIE efficiency up to ~81% at an optical density of 10 kWm^−2^. Based on the aforementioned excellent properties, research in the PIE field frequently makes use of carbon-based materials.

#### 3.1.4. Composite Materials

**Figure 7:**
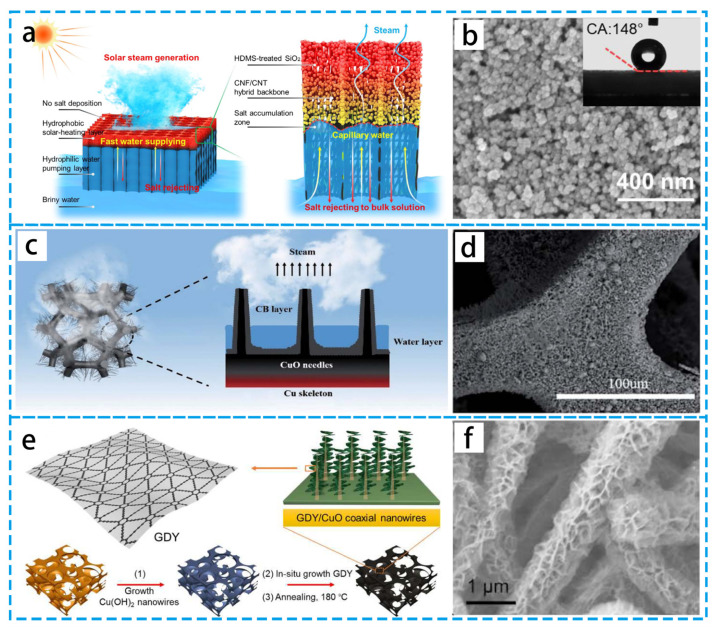
(**a**) Schematic diagram of Janus wettability PIE evaporator. (**b**) High-magnification SEM image of SiO_2_ nanoparticles. (**c**) Schematic diagram of the evaporation behavior of CuO/Cu-CB foam. (**d**) SEM image of CuO/Cu-CB foam. (**e**) Schematic diagram of GDY-based hierarchical structure. (**f**) SEM image of GDY/CuO coaxial nanowires cladding copper foam. (**a**,**b**) Reproduced from Ref. [68]; (**c**,**d**) Reproduced from Ref. [69]; (**e**,**f**) Reproduced from Ref. [70].

High solar radiation band absorption and low infrared band emissivity are characteristics of the ideal solar absorber. Metallic nanoparticles, semiconductor materials, and carbon-based materials are the primary photothermal materials. Another method to increase absorption is the synthesis of hybrid materials. To increase the photothermal conversion efficiency, numerous hybrid material assemblages have been produced. These materials gain complimentary and synergistic optical capabilities by utilizing the optical characteristics of distinct materials. As demonstrated in Figure 7a,b, Wang’s group [68] reported a Janus wettability PIE evaporator based on a hybrid SiO_2_/cellulose nanofiber/CNTs. The hydrophobic component brings the evaporator layer out of the water for better thermal localization; the hydrophilic component for continuous pumping allows the Janus PIE device to have excellent evaporation properties. As seen in Figure 7c,d, Xu’s group [69] prepared a functionalized CuO/Cu-CB foam based on Joule heating via the photothermal effect that can use an all-day PIE in low light. This CuO/Cu-CB foam can achieve an evaporation rate of 4.5 kg h^−1^m^−2^ when the voltage is as low as 2 V. A multidimensional architecture based on graphdiyne (GDY) [70] was created for effective solar steam power generation (Figure 7e,f). The purpose of this multidimensional structure is to make it easier to reduce surface reflection, improve optical absorption, and increase a significant amount of specific surface area for heat exchange. The majorization of material composition and structure is the key to enhancing solar vapor efficiency. The optical qualities of the absorption range and intensity can be considerably improved by combining different solar-absorbing materials while keeping the synergistic effect of their respective inherent optical and structural properties, leading to efficient photothermal conversion.

The current focus of research and development is on novel designs of unique nanomaterials to solve the problems associated with attaining high-efficiency, solar-driven vapor production. Table 1 summarizes a variety of nanomaterials for desalination or freshwater production in terms of the solar irradiance and evaporation rate. It can be seen that an excellent PIE performance can be achieved with a variety of different types of materials through rational design of the material structure. However, in terms of the cost and environmental friendliness, carbon materials are still the better choice.

### 3.2. Thermal and Water Management

The usage of thermal energy, both at the micro and macro levels, will be significantly impacted by the thermal management of the photothermal conversion system. Macroscopic thermal management entails the use of insulation materials to prevent heat loss [71]. And microscopic thermal management can optimize the photothermal conversion performance of PIE systems [72]. It is crucial to guide the thermal energy to the target component since the PIE absorber and surroundings are exchanging heat [73]. Thus, excellent thermal management determines that the heat generated by the PIE materials can be utilized as much as possible for interfacial evaporation.

**Figure 8:**
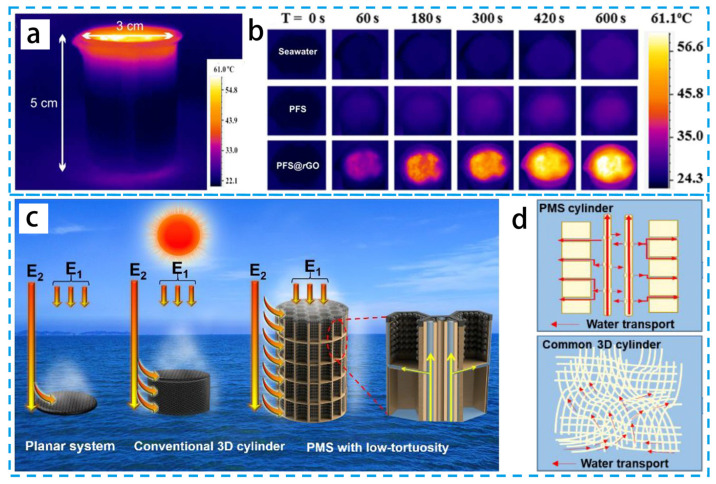
(**a**) Infrared images of the corresponding temperature gradient distribution. (**b**) Infrared images of the temperature of PFS@rGO, PFS, and seawater. (**c**) Schematic diagrams of planar systems, cylinders with tortuous pores, and low-curvature PMS solar energy utilization and schematic diagrams of water transport processes plotted on PMS and ordinary 3D columns, (**d**) schematic diagrams and IR images. (**a**,**b**) Reproduced from Ref. [79]; (**c**,**d**) Reproduced from Ref. [80].

Thermal conduction, thermal convection, and thermal radiation are the three basic ways of heat loss [74,75,76]. The direct-contact mode is where the membrane floats on the water surface; thermal conduction is most common in this system [77]. In order to solve this problem, an indirect-contact structure design is increasingly widely used for efficient interfacial evaporation [78]. In terms of reducing the heat loss, the microstructure and surface wettability of the nanofiber membrane have great impacts on temperature management. Some researchers believe that hydrophilic surfaces affect the distribution of liquid at the gas–liquid interface, causing a thin film of water to form on the three-phase contact line, which in turn promotes evaporation. Through the above scheme, the black plant fiber sponges’ (PFS@rGO) average absorption efficiency is about 95.5% after subtracting the corresponding transmittance (0.5%) and reflectance (4.0%) [79], as shown as Figure 8a,b. The partially reduced graphene oxide and porous design of PFS@rGO also provide significant thermal insulation qualities. Thermal localization is important to improve the efficiency of evaporators, and the thermal localization structure design has inspired many further studies.

The water-supply method affects both thermal management and evaporation sustainability, thus affecting evaporation rates [81,82,83]. A suitable water content of the interfacial photothermal material causes a high evaporation rate. The structure of maize straw (MS) has microchannels with a low curvature. These straight microchannels act as the main arteries of the plant, transporting water from the soil to a height. Some research prepared MS with polypyridine (PPy) (denoted as PMS) as a 3D PIE [80], as shown as Figure 8c,d. The simultaneous action of the direct microtubular structure allows effective vertical capillary water lifting and horizontal water transport through the microgap to the outer surface. The actual desalination steam generation rate of the optimized PMS array prototype under natural conditions is 2.2 L m^−2^ h^−1^.

### 3.3. Anti-Salt Strategy

To date, the treatment of highly concentrated waste brine (C_NaCl_ > 7 wt%) with minimized energy consumption has been considered the most challenging task [84]. Salt accumulation is one of the main obstacles to large-scale industrial applications. During the PIE process, salt ions accumulate to form concentration polarization, and the local salt concentration (C_a_) increases significantly. Once the local salt concentration is oversaturated (C_a_ > C_sat_; C_sat_ is the saturation concentration of salt), the salt can crystallize inside or on the absorber, leading to degradation of the device’s performance and a shortened lifetime. Therefore, regulating the local salt concentration is the key to prevent salt accumulation [85].

**Figure 9:**
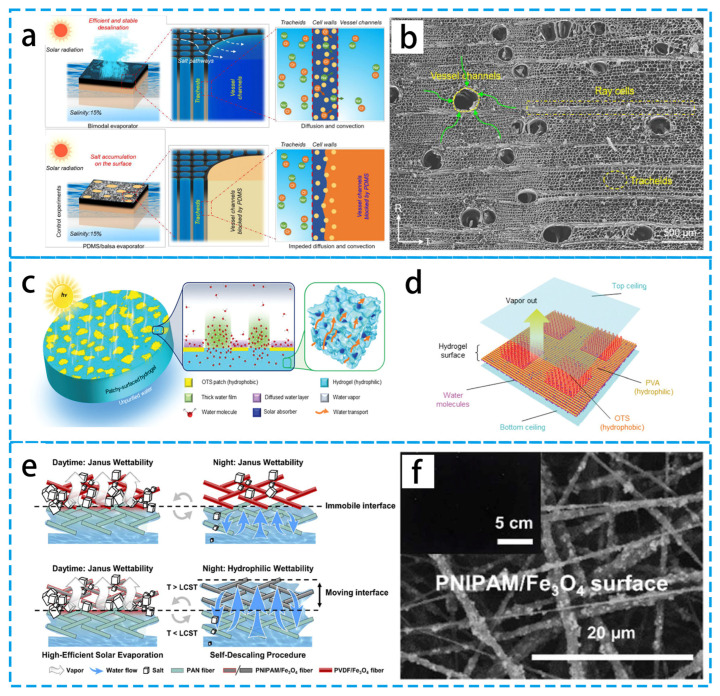
(**a**) Microstructure and working principle of balsa. (**b**) SEM image of porous balsa wood. Balsa wood has many wider ductal channels and narrower tubular cells. (**c**) Schematic diagram of the hydrogels (PSHs) used for enhanced photothermal evaporation. (**d**) MD simulation of PSHs simulating a sandwich-like model of the hydrogel’s patchy surface. (**f**) SEM image of the PNIPAM/Fe_3_O_4_ surface. (**a**,**b**) Reproduced from Ref. [87]; (**c**,**d**) Reproduced from Ref. [89]; (**e**,**f**) Reproduced from Ref. [90].

Fundamentally, if the concentration grows, salt crystallization will unavoidably happen when water evaporates [86]. A reliable and effective solar steam generator for the desalination of high-salinity seawater was prepared by Hu’s group [87] using balsa wood (Figure 9a,b). Through transport between microchannels and macrochannels, as well as rapid capillary transport within microchannels, the inherent bimodal porous interoperable microstructure can quickly replenish the surface-evaporated brine. The PIE evaporator provides excellent stability for high-salinity brines. In addition, Zhu’s group proposed to design a salt-tolerant PIE evaporator based on the Donnan effect [88], which has the ability to limit the salt tolerance of sodium ions. The high chemical potential of confined Na^+^ leads to a Donnan distribution balance, which minimizes the diffusion of salt ions into the water supply layer and thus, fundamentally avoids salt accumulation. This Donnan-effect-based strategy provides a new solution for photothermal evaporation to treat high-salinity seawater.

In recent years, in response to the salt-accumulation problem, researchers have found that regulating the surface wettability of photothermal systems [91], separating water evaporation (hydrophobic region) from water transport (hydrophilic region), is another scheme to regulate the local salt concentration. As shown in Figure 9c,d, Yu’s group [89] demonstrated a hydrophilic hydrogel evaporator with hydrophobic island patches that was able to achieve a high evaporation mass. This exceptionally high rate is due to the synergistic effect of the two wetting regions. Molecular dynamics simulations provide consistent results, where inhomogeneous surface wetting properties modulate the escape behavior of water molecules and thus accelerate evaporation. A self-descaling Janus evaporator (SJE) was fabricated for the long-lasting and effective desalination of high-salinity seawater [90], as shown in Figure 9e,f. The evaporator consists of Fe_3_O_4_ embedded with a poly(N-isopropylacrylamide) (PNIPAM) nanofiber layer and hydrophilic polyacrylonitrile (PAN) nanofiber layer. Under sunlight, the top surface separates from the evaporative interface (i.e., the air–water interface), preventing salt accumulation and facilitating efficient solar vapor production. At night, the air–water interface moves to the upper surface. The evaporator achieves a long-term stable evaporation of 20 wt% concentration of brine within five days. Thus, regulating the surface wettability of the photothermal system is an effective way to solve the salt accumulation on the PIE evaporator.

As an important branch of PIE materials, nanofiber materials have received extensive attention from researchers due to its simple preparation, rich variety, and strong scalability. Compared with nanofiber materials, traditional porous materials are time-consuming and complicated and usually have a lower specific surface area, poor elasticity, and poor flexibility. In contrast, nanofiber materials with flexibility, light weight, easy preparation, and durability are convenient for large-scale applications [41]. Due to the high aspect ratio of one-dimensional nanofiber membranes, the increased specific surface area and high porosity of the nanofiber membrane significantly improves the reactivity and selectivity, so that nanofibers can be combined with nanomaterials of different dimensions, and have various synergistic effects, interface effects, etc., demonstrating perfect functionalization [33]. More importantly, for PIE, a super-high specific surface area can provide a large evaporation area, which means a high evaporation rate. In addition, nanofibrous membranes have interlaced porous structures and exhibit super-high porosity, not only enhancing the light absorption due to the multiple reflections of light and providing channels for vapor escape, but also preventing, to a certain degree, heat loss and increasing evaporation efficiency. Therefore, nanofiber composites have been good candidates for PIE materials and shown promising application prospects in solar desalination.

## 4. Photothermal Evaporation of Electrospun Nanofibers

Electrospinning technology provides the means to produce fiber materials in the micrometer to nanometer range, allowing the controlled adjustment of the microstructure and chemical composition of the fibers to achieve properties superior to those of bulk materials or materials of the same size [92,93,94]. The main advantage of electrospun materials is the plasticity of the fibers, which can both form flexible two-dimensional film materials and construct controllable 3D aerogel materials with porosity [95]. This paper introduces several types of fiber-based materials for photothermal evaporation and reviews the relationship between material structure and evaporation performance to provide a reference for PIE.

### 4.1. Two-Dimensional Membrane Materials

Phase transformation [96], sol-gel, and freeze-drying [97], which are typically time-consuming, have low specific surface areas, and have poor elasticity and flexibility, can be used to prepare conventional porous membranes. In contrast, electrospun nanofibers are flexible, lightweight, easy to prepare, and durable for large-scale applications [98,99]. The unique microstructure facilitates multiple reflections of light within the membrane. Based on the above advantages, electrospun nanofibers have great prospects for application in PIE.

#### 4.1.1. Multi-Structure Design of Single Fibers

**Figure 10:**
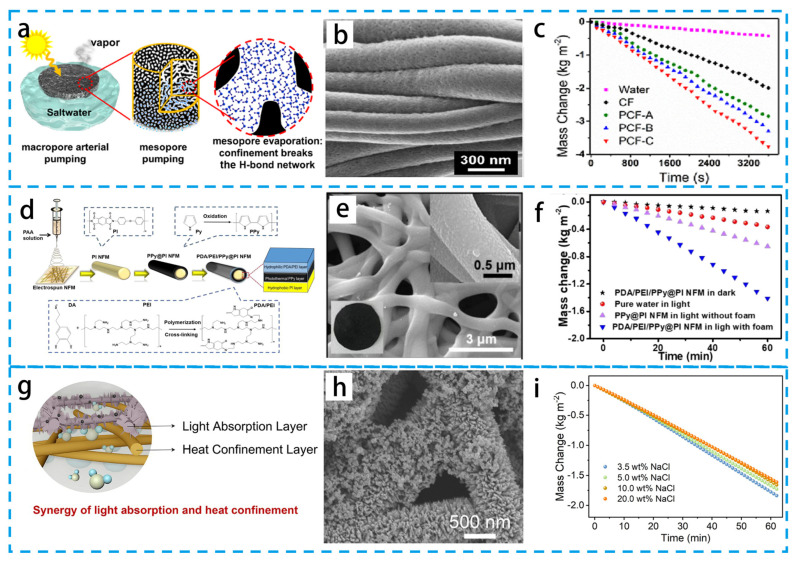
(**a**) Schematic diagram of porous carbon fibers. (**b**) SEM image of the external surface of PCF-B. (**c**) Evaporation mass over time under one solar irradiation. (**d**) Schematic diagram of the fabrication process of nanofiber membranes. (**e**) SEM images and corresponding optical images of PDA/PEI/PPy@PI NFM. (**f**) Comparison of the mass change under four different conditions. (**g**) Schematic diagram of the structure and function of Janus interfacial solar vapor generation (J-ISVG). (**h**) SEM images of C@CuO films. (**i**) Evaporation rate of C@CuO-J60 evaporator at 1/2/3 sun. (**a**–**c**) Reproduced from Ref. [106]; (**d**–**f**) Reproduced from Ref. [107]; (**g**–**i**) Reproduced from Ref. [108].

Electrospinning can prepare fiber materials with a variety of multilevel structures, such as core–shell structure fibers [100], porous structure fibers [101,102], hollow structure fibers [103], multichannel structure fibers [104], and beaded structure fibers [105]. This provides unlimited possibilities for the application of fiber materials in PIE. As shown in Figure 10a–c, Liu’s group [106] investigated the latent heat reduction using block copolymer-based fibers, which achieved PIE rates of up to 3.8 kg m^−2^ h^−1^ over the entire solar spectrum and pore-bound water evaporation enthalpy reduction. The significantly higher evaporation rate for mesopores compared to micropores is due to the enthalpy reduction and water transport caused by nanoscale confinement. This study provides nano-constrained effects that can help the further design of porous materials.

Wang’s group [107] prepared a durable and corrosion-resistant fiber membrane with a core–shell structure by coating PPy on the surface of polyimide (PI) fibers followed by polydopamine (PDA)/polyethyleneimine (PEI) co-deposition. As shown in Figure 10d–f, the core–shell structure of a PDA/PEI/PPy@PI nanofiber membrane has broadband solar absorption and 30 days of continuous corrosion resistance. In addition, the nanofiber membrane effectively prevents swelling and maintains excellent mechanical strength even under wet conditions. As shown in Figure 10g–i, Zhao’s group [108] prepared a Janus PIE evaporator based on hydrophilic carbon black decorated copper oxide (C@CuO) film and hydrophobic polymer nanofibers. The C@CuO surface reduced the evaporation enthalpy and the Janus PIE evaporator achieved a high evaporation rate of 1.88 kg m^−2^ h^−1^. This synergistic engineering of ultra-low heat loss offers a bright future for efficient PIE application.

The same strategy can be used for other types of glass and fabric fiber materials. The layered structure (NiCo_x_S_y_-PANI@GF) [109], where a large number of NiCo_x_S_y_ nanosheets are grown on polyaniline (PANI) modified glass fiber (GF) membranes, rationalizes the development of a flexible, floating, and efficient photothermal interfacial water evaporation device. Wang’s group [110] developed Ti_4_O_7_ nanofiber membranes with synergistic photothermal and electrothermal effects for PIE. Chen’s group [111] fabricated a layered PAN@CuS and presented an evaporator prototype. The water evaporation enthalpy of the layered PAN@CuS fabric was significantly lower compared to pure water due to the disorder of hydrogen bonding at the CuS interface. Under one solar irradiation, the model exhibits high evaporation rates and a high concentration of seawater desalination without solid salt crystallization, allowing for further photothermal evaporation.

Recently, for the design of single fibers, multi-structure fibrous materials such as hollow fibers, core–shell fibers, multichannel fibers, multicavity microcapsules, side-by-side fibers, vesicle fibers, and lotus-root-like fibers can be controllably prepared using electrospun technology. This multi-structure fibrous materials can realize fine control of the PIE process on the nanometer and micrometer scales, which provides a new idea for the development of new high-performance PIE devices. However, the major difficulty in the current design based on a single fiber is that it cannot be prepared on a large scale.

#### 4.1.2. Multi-Structure Design of Fiber Membrane

**Figure 11:**
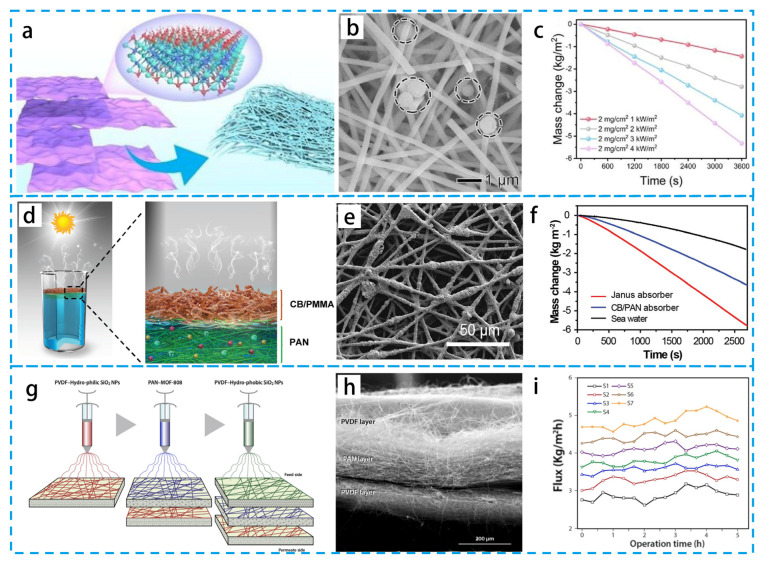
(**a**) Schematic diagram of the fabrication of the photothermal Al_2_O_3_/TiO_2_/Mxene membrane. (**b**) SEM image of the photothermal flexible Al_2_O_3_/TiO_2_/Mxene membrane (**c**) Variation of seawater mass of 2D Al_2_O_3_/TiO_2_/Mxene-2 membrane under different solar light intensities. (**d**) Schematic diagram of PIE and seawater desalination. (**e**) SEM image of carbon black nanoparticles deposited on PMMA/PAN. (**f**) Variation of seawater mass with Janus absorber, with CB/PAN membrane, and without any absorber. (**g**) Schematic diagram of the start of the three-layer preparation procedure. (**h**) Optical image of PVDF/PAN/PVDF. (**i**) Fluxes of every sample. (**a**–**c**) Reproduced from Ref. [112]; (**d**–**f**) Reproduced from Ref. [114]; (**g**–**i**) Reproduced from Ref. [115].

The layer structure of fiber membranes refers to the number of layers of functional membrane materials designed according to the actual needs, such as single-layer super-impregnable membranes, double-layer asymmetric impregnable membranes, sandwich-structure membranes, etc. The single-layer structure is usually designed by adding or attaching photothermal materials inside or on the surface of its fibers. As shown in Figure 11a–c, Dai’s group [112] prepared flexible 2D Al_2_O_3_/TiO_2_/MXene membranes with good mechanical properties using a simple drop-casting method. Based on this unique membrane structure, the evaporation rate was as high as 1.43 kg m^−2^ h^−1^. Zheng’s group [113] introduced a polycaprolactone nanofiber composite fiber membrane containing carbon nanotubes or carbon nanoparticles with an average absorbance of 0.94 and 0.93 at visible and near-infrared wavelengths, respectively, making it an excellent broad-spectrum solar absorber. This demonstrates a new method of preparing fiber interfacial evaporators from degradable biocompatible materials and carbon materials, showing great application prospects.

Due to its structural limitations, the single-layer photothermal evaporator has a single function. For this reason, double-layer asymmetric immersion evaporators have significantly improved the evaporation efficiency due to their lower hydrophilic and upper hydrophobic structure, which facilitates thermal localization, water transport, and vapor escape. Li’s group [116] designed a multifunctional asymmetric immersion Janus membrane for all-day freshwater harvesting. The membrane exhibited an excellent performance with a PVA/PAA interlayer coating, and a high-quality freshwater supply was achieved using the membrane with excellent solar desalination. As shown in Figure 11d,e, the flexible Janus PIE absorber is prepared using sequential electrospinning [114]. This Janus structure achieved the effect of absorbing light from the upper hydrophobic CB nanoparticle-coated polymethyl methacrylate (PMMA) layer and pumping water from the lower hydrophilic PAN layer. Janus PIE absorbers exhibit high efficiency and a stable water output.

The surface chemistry (or wettability) also has a significant effect on the PIE performance of water-surface independent bilayer films. The designed evaporation system consists of an upper plasma photothermal conversion layer and a lower porous support layer [117]. The induced plasma heat is localized within the film and the wettability of the evaporation system is regulated by controlling the surface chemistry, differentiating between hydrophilic and hydrophobic anodes. Rather than the wettability of the top photothermal conversion layer, the rate of evaporation is primarily determined by the bottom support layer.

Christopher Q. proposed a three-layer nanofiber membrane consisting of polyvinylidene fluoride (PVDF) blended with hydrophobic SiO_2_ NPs, PAN/metal organic frameworks (MOFs), and PVDF blended with hydrophilic SiO_2_ NPs [115], as shown in Figure 11g–i. This new film effectively produces drinking water that meets and exceeds drinking water standards in terms of electrical conductivity. To further improve the energy efficiency, Chen’s group [118] developed a graphene/polyimide (LIG/PI) photothermal film with a multilayer structure through electrospun technology for fast and efficient PIE. The multilayer structure of the LIG/PI film increases the evaporation area and reduces the energy loss due to diffuse reflection of light in the thermal insulator and pump channel. The sandwich structure was also utilized by Chen’s group [119] to prepare a novel sandwich Janus membrane. PDA/polyethyleneimine (PEI) was wrapped on one side of a PVDF microporous membrane, and then a hydrophobic nanofiber layer was constructed by spinning on the other side of the membrane as a moisture-resistant layer. The permeate flux changes in the MD membrane were verified and predicted with numerical simulations, which, combined with the experimental results, revealed the superiority of the sandwich.

Currently, membrane materials with layer structures on the micrometer scale, such as the aforementioned Janus structure, sandwich structure, and gradient structure, are the most studied of the PIE materials. These functional layer structures are crucial in water transport, vapor transport, and efficient energy utilization. Electrospinning technology is one of the simplest and most effective methods to prepare such materials. However, fine control of the relative proportions of each functional layer in the fiber membrane is still the main subject of further research in the future.

### 4.2. Three-Dimensional Materials

Hydro/aerogels are usually composed of a cross-linked hydrophilic polymer network and many water molecules with a large internal surface area, high water content, and high reusability [120,121,122]. In recent years, hydro/aerogels have been applied as a new technological design for solar evaporators due to their adjustable internal water distribution (degree of cross-linking), and hydro/aerogel materials are also considered as one of the most important materials for water evaporation and desalination processes [123,124]. In addition, embedding nanomaterials into hydrogels facilitates the improvement of some properties of hydrogels, making them more suitable for rapid vapor production compared to other materials.

**Figure 12:**
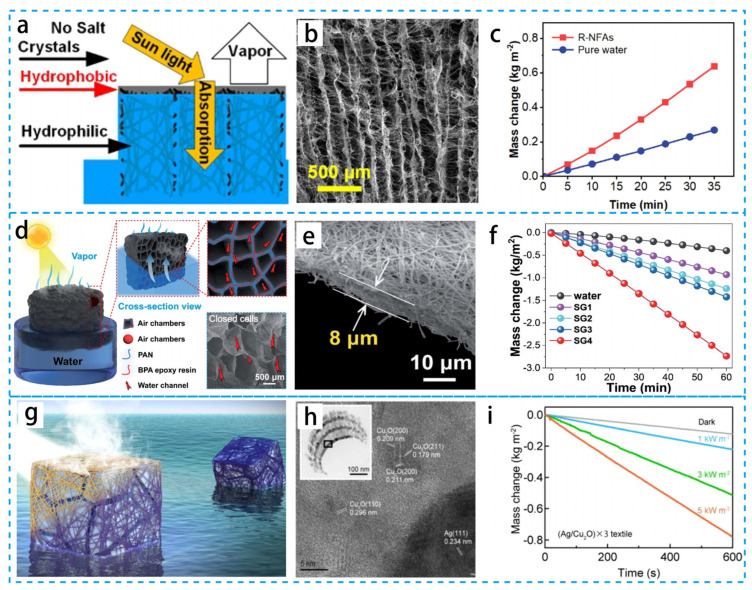
(**a**) Sunlight absorption, water transport, vapor production, and salt resistance of reed leaves. (**b**) Graded porous microstructure of R-NFAs. (**c**) Cumulative mass loss of R-NFAs and pure water over time at one sun. (**d**) Schematic, morphology of the 3D composite foam. (**e**) Cross-sectional scanning electron microscopy images. (**f**) Corresponding variation of water mass with time. (**g**) Fast solar heating of antimicrobial silver and Cu_2_O nanostructured plasma textiles for clean water production. (**h**) TEM image of (Ag/Cu_2_O) × 3. (**i**) Temporal profiles of the mass change of (Ag/Cu_2_O) × 3 textiles under different solar light intensities. (**a**–**c**) Reproduced from Ref. [127]; (**d**–**f**) Reproduced from Ref. [128]; (**g**–**i**) Reproduced from Ref. [129].

Benefitting from the rich porous network, low thermal conductivity, and high porosity, light absorbers with hydro/aerogels and foams as substrates are among the ideal materials for the preparation of PIE devices [125,126]. The irregular network structure and randomly distributed pores in these 3D materials can achieve a rapid transport of liquid water under restricted-domain conditions, while avoiding large heat dissipation and thus improving the evaporation efficiency. As shown in Figure 12a–c, Deng’s group [127] designed biomimetic graded nanofiber aerogels with parallel-arranged microtubules and efficient hydrophobic surfaces. This foldable tube wall enables reed leaf nanofiber aerogels (R-NFAs) to have excellent mechanical properties. R-NFAs can effectively absorb sunlight and evaporate salt water into vapor at a rate of up to 1.25 kg m^−2^ h^−1^. More importantly, R-NFAs can work stably in 26.3 wt% brine, showing good PIE application. As shown in Figure 12d–f, Xu’s group [128] developed a 3D polymer foam in a monolithic interfacial vapor generator. This foam uses a large number of gas capsules separated by mesh-like hydrophilic nanofibers. The polymer composite, with a low thermal conductivity, ultra-light weight, and efficient water expansion, is one of the few interfacial vapor generators that integrate various interesting properties into a monolithic polymer foam for high-performance solar energy. Shougo Higashi’s group [129] reported a freestanding fabric consisting of interconnected Cu_2_O and Ag NPs, as shown in Figure 12g–i. This fabric has antimicrobial properties, which would reduce the risk of steam-induced bacterial transfer during long-term intermittent use and the cost of subsequent water disinfection. Tang’s group [130] designed a 3D fiber aerogel that floats on the water surface and continuously self-pumps water. More notably, aggregation-induced emission (AIE) photothermal molecules were doped in the 3D fiber aerogel, conferring on it a superior ability to convert solar energy into heat. Combining these unique advantages, the proposed 3D fiber aerogel has a high evaporation rate under natural sunlight irradiation. This 3D fiber aerogel could provide a new strategy for seawater desalination. Wang’s group [131] developed a novel composite membrane for oil contamination in MD. The composite membrane consists of a polytetrafluoroethylene (PTFE) hydrophobic backing and a hydrophilic polyvinyl alcohol/silica nanoparticle (PVA-Si) hybrid fiber coating prepared with sol-gel and electrospinning. The superhydrophobic coating can effectively alleviate oil scaling in MD, and the prepared composite membrane can enable MD desalination of highly saline wastewater containing high concentrations of hydrophobic contaminants. Ma’s group [132] demonstrated a nanofiber hydrogel–reduced graphene oxide (NHrG) membrane. By monitoring the evaporation enthalpy, it was demonstrated that the evaporation of intermediate water dominates the interfacial evaporation process and, therefore, plays a key role in reducing the evaporation enthalpy. The evaporator showed an excellent desalination performance in the treatment of freshwater. The water/aerogel shows that functional materials and water are bonded at the molecular level, and polymers with solar energy absorption can transfer the collected energy directly to water molecules, facilitating thermal localization and thus having unique advantages for PIE.

Three-dimensional fiber-based aerogel materials have demonstrated excellent performance in the field of interfacial evaporation because of their ability to achieve high evaporation efficiencies beyond the theoretical limit. In the future, mechanisms to enhance the evaporation efficiency, such as the absorption of ambient energy and reduction in the enthalpy of evaporation of liquid water, should be explored in depth.

Some typical examples belonging to different structures are listed in Table 2. By comparing the parameters, including the design strategies, characteristic evaporation rate, and light density, it can be concluded that each of these materials has its own characteristics. It can be seen that an excellent PIE performance can be achieved through rational design of the material structure. The choice of design strategy should be considered according to the practical application scenario. For example, during PIE processes, there is a need for antimicrobials, which can be targeted at single fibers for loading functional drugs; wettability can be used to reduce the problem of salt crystallization, which can be targeted at fiber membranes for modification; to maximize evaporation efficiency, 3D materials can be designed to use environmental energy to promote interfacial evaporation.

### 4.3. Derivative Applications in Photothermal Evaporation of Electrospun Fibers

In recent years, we have seen numerous developments in PIE based on electrospun fiber materials [133,134,135]. Many fiber materials have also demonstrated significant roles in energy conversion, photothermal catalysis, and wastewater treatment in addition to the photothermal evaporation process, considerably expanding the range of applications for fiber-based photothermal evaporation devices.

#### 4.3.1. Energy Conversion and Photothermal Catalysis

Recently, solar energy has been used extensively to produce other forms of energy [136]. Water-wave energy, concentration gradients, and heat from the liquefaction of water vapor are just a few examples of the additional energy that can be converted into electrical energy during the interfacial evaporation process [137,138]. To produce electricity and freshwater simultaneously, energy storage has recently been integrated with solar evaporation techniques. A significant concentration gradient between the water at the interface and the water in the bulk phase is created by the rapid evaporation of water from the surface of the photothermal evaporation device, but this has received little attention.

**Figure 13:**
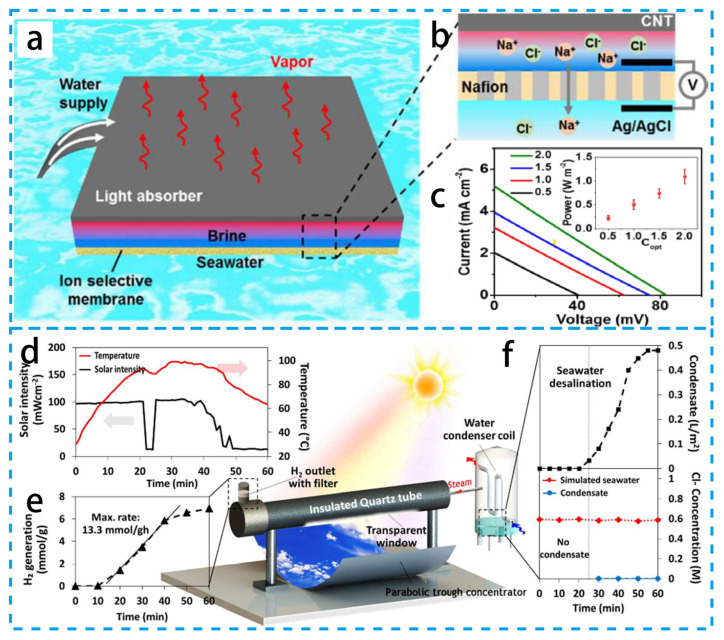
(**a**) Schematic diagram of the hybrid system for solar desalination and salinity power extraction. (**b**) Mechanism of salinity difference power generation. (**c**) Current–voltage curves of the hybrid device under solar irradiation. (**d**) Schematic diagram of the prototype reactor. (**e**) Hydrogen production of prototype reactor. (**f**) Condensate volume and chlorine concentration of prototype reactor. (**a**–**c**) Reproduced from Ref. [139]; (**d**–**f**) Reproduced from Ref. [10].

As shown in Figure 13a–c, Zhou’s group [139] demonstrated that the theoretical real-time salinity power generated between the interfacial water and the bulk seawater during vapor production under one solar illumination can reach 12.5 W m^−2^. By using a hybrid system based on a commercial Nafion membrane and carbon nanotube-modified filter paper, a solar illumination of an additional ~1 W m^−2^ of electricity was obtained from a single solar exposure. As illustrated in Figure 13d–f, Ghim Wei Ho’s group [10] developed a solar collector based on a nano-SiO_2_/Ag@TiO_2_ core–shell composite that enabled the desalination of seawater and catalytic hydrogen production from seawater. Surface-dominated catalytic reactions and vapor-production processes are directly triggered by the photothermal effect produced by plasmonic metal nanoparticles with the least amount of energy loss. Excellent stability is displayed by the solar collector in both light and marine environments. This finding demonstrates the viability of low-cost, sustainable photothermal-driven desalination and catalysis, and it holds enormous promise for raising energy and water production. Wang’s group [131] developed a novel composite membrane for oil contamination in MD. The composite membrane consists of a polytetrafluoroethylene (PTFE) hydrophobic backing and a hydrophilic polyvinyl alcohol/silica nanoparticle (PVA-Si) hybrid fiber coating prepared using sol-gel and electrospinning. The superhydrophobic coating can effectively alleviate oil scaling in MD, and the prepared composite membrane can enable MD desalination of highly saline wastewater containing high concentrations of hydrophobic contaminants. Nanofiber combined functional materials have wide promise for energy conversion and photothermal catalysis.

#### 4.3.2. Wastewater Treatment

Fibrous materials loaded with bacteriostatic components, such as Ag nanoparticles, CuO nanoparticles, etc., are excellent candidates for multifunctional photothermal materials [140] because they not only perform effective photothermal conversion but also have potent bacteriostatic properties. Under typical conditions, many bacteria are invariably present in water [141]. As previously shown in Figure 10g–h, the Janus evaporator prepared by Hou et al. [108] utilized a hydrophilic C@CuO film as a light-absorbing layer and hydrophobic polymeric nanofibers as an insulation layer. Additionally, due to antibacterial impact, the antibacterial efficacy of C@CuO against Gram-positive Staphylococcus aureus and Gram-negative Escherichia coli was enhanced. Both the blank controls showed typical bacterial multiplication following a 24 h incubation on agar plates with or without light irradiation. On the other hand, no bacterial activity was seen on C@CuO agar plates, whether they were exposed to light or not. This is because copper ions could be absorbed by spores as they germinated, killing the bacterial colonies.

**Figure 14:**
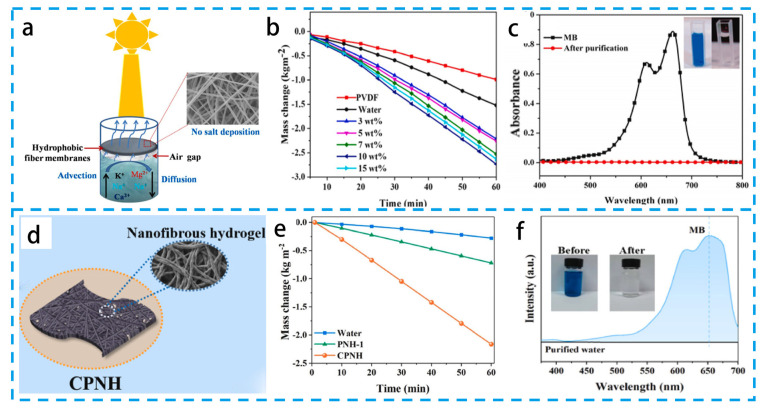
(**a**) Schematic diagram of hydrophobic fiber membrane. (**b**) Cumulative mass loss of fiber membranes, (**c**) Color of MB before and after sunlight irradiation. (**d**) Schematic diagram of CNTs@PVA nanofiber hydrogel preparation. (**e**) SEM images of the CPNH. (**f**) UV-Vis spectra of organic dyes before and after the solar desalination system. (**a**,**b**) Reproduced from Ref. [145]; (**c**,**d**) Reproduced from Ref. [146].

By raising the temperature to hasten the evaporation of water, pure liquid water was collected from water contaminated with organic matter or heavy metal ions [142,143,144]. Graphite carbon nitride/tungsten oxide heterochemicals (Cs_0.32_WO_3_@g-C_3_N_4_) were effectively synthesized by Wu’s group [145] using a straightforward solvothermal approach (Figure 14a–c). The heterochemicals were electrospun into Cs_0.32_WO_3_@g-C_3_N_4_/PVDF fiber membranes after being doped into PVDF. Using photothermal fiber membranes, water evaporation, seawater desalination, and wastewater treatment were studied. The highest temperatures measured in NIR and total solubility were 85 °C and 90 °C, respectively. The membranes exhibited excellent resistance to salt contamination, with PIE efficiency and salt removal rates of 95.4% and 99.9%, respectively. In addition, electrospun fibers were effective in the removal of organic dyes, 4-nitrophenol, and tetracycline. These excellent performances are attributed to the hydrophobic nature of PVDF, which allows the formation of an air gap between the water and the fiber membrane. The air gap selectively allows water vapor to enter the fiber membrane, while salt and organic contaminants diffuse back into the bulk water, which is the key to successful desalination. This paves the way for the creation of novel multipurpose fiber membranes for desalination and wastewater treatment in general. Although solar desalination is regarded as one of the sustainable and environmentally benign solutions to the world’s freshwater shortage, more study is still required, particularly on the purification of non-VOC-contaminated water sources. By using electrospinning and chemical cross-linking techniques, Fan’s group synthesized CNTs @ polyvinyl alcohol (PVA) nanofiber hydrogel (CPNH) evaporators [146] (Figure 14d–f). Due to their superior light-absorbing capabilities, the CNT evaporators displayed a high light-absorption performance (90%) across the whole spectral range (250–2500 nm). The developed evaporator can achieve a PIE rate of 2.16 kg m^−2^ h^−1^ due to the linked holes created using electrospinning and the intermediate water in the hydrogel. The CNTs@PVA nanofiber hydrogel evaporator also offers great salt resistance, durability, and outstanding self-cleaning capabilities. The PAN@CoMn-LDH (LDH = layered double hydroxide) membrane with hierarchical structure was synthesized using electrospinning followed by in situ growth and a template-etching strategy [147]. The evaporation rate of the PAN@CoMn-LDH membrane for wastewater was 3.09 kg m^−2^ h^−1^. The concentrations of Cd^2+^, Cr^3+^, Ni^2+^, and Pb^2+^ after purification decreased from 137.1, 120.5, 132.3, and 87.4 to 0.000532, 0.0026, 0.00432, and 0.00439 mg L^−1^, respectively, which reach the drinking water standards of the World Health Organization (WHO). This study provided a new facile design strategy for the development of outstanding photothermal conversion materials applied in solar-driven evaporation that shows broad prospects in seawater desalination.

Fiber-based evaporators have shown excellent performance in removing heavy metal ions and purifying non-volatile organic pollutants, opening up new avenues for the development of high-performance evaporators for seawater desalination and wastewater purification. However, in the case of wastewater containing volatile organic compounds, the evaporator can only obtain non-compliant products, which is a major challenge for the PIE equipment.

## 5. Summary and Prospect

In this review, the photothermal conversion mechanism, thermal management, water transport, and salt-resistance schemes of various photothermal materials in PIE are presented in the first section. The second part of this paper describes methods for increasing the light absorption of materials, optimizing the solar thermal conversion efficiency, reducing heat loss, improving thermal localization performance, and designing efficient fluid transport. This provides a theoretical reference for the design of high-efficiency PIE systems. This paper also introduces several types of fiber-based materials for photothermal evaporation and reviews the relationship between the material structure and evaporation performance. Finally, we present electrospun fiber materials for interfacial evaporation, which also play an important role in energy conversion, photothermal catalysis, and wastewater treatment, greatly expanding the range of applications for fiber-based PIE. The aim of this review is to provide direction for the design of PIE devices by effectively understanding the photothermal conversion and thermal mass-transport mechanisms of multi-structural fiber materials.

The rapid and remarkable development of solar evaporative photothermal materials has greatly boosted research in this field. However, there are some key challenges to be faced.

(1) The gap between the current state of the art and practical applications is significant. This gap encompasses all aspects, including system design and reliability to enable effective condensation and water collection, as well as the long-term material stability and durability of real water sources (such as oceans, natural freshwater, and industrial wastewater). Other aspects to consider include the impact of external variables such as wind, sporadic sunlight, water volume, salt scaling of solar absorbing materials, and the elimination of volatile organic chemicals found in the water source. Therefore, more work is needed to develop durable solar photothermal materials with good thermal/chemical stability, recyclability, and compatibility with a variety of environments, as well as to design effective prototypes in terms of latent heat recovery, minimization of heat losses, and compact installations that significantly affect the overall efficiency and yield.

(2) Evaporation at the photothermal interface is a particularly complicated process due to strategies to increase the water supply, decrease thermal management, and prevent salt precipitation, which may be in conflict with one another. The best way to strike a balance between these three factors is a crucial issue.

(3) The kinetics and thermodynamics of water molecule evaporation, heat conduction, and water transport are still not fully understood in terms of the mechanism, which is a crucial area for future advancement in the discipline of electrical engineering. On this basis, the design of PIE systems that exceed the conventional theoretical limit of evaporation efficiency by absorbing ambient heat or reducing the evaporation enthalpy of water will be the subject of future research.

Although some challenges remain, electrospun nanofibers with their unique structural advantages to improve photothermal conversion efficiency are promising technologies for preparing photothermal interfacial evaporators. This review is expected to stimulate more related research and promote the application of electrospun nanofiber materials for excellent performance in PIE.

## Figures and Tables

**Table 1:** Evaporation performance based on different types of photothermal materials.

Type	Photothermal Material	Substrate	Solar Irradiance (kW m^−2^)	Evaporation Rate (kg m^−2^ h^−1^)	Ref.
Carbon materials	CNTs	PAN fiber	1	1.28	[24]
Composite materials	CoO_x_/CB	Carbon fiber	1	3.23	[45]
Nanometallic materials	Au nanorod	CNTs	1	1.85	[50]
Carbon materials	CNTs	SiO_2_	1	1.31	[51]
Carbon materials	Graphene	Graphene foam	1	2.60	[52]
Semiconductors	Black TiO_2_	PVDF	~1	1.13	[54]
Carbon materials	rGO	PU	10	11.24	[55]
Semiconductors	CuS	PAN fiber	-	-	[59]
Carbon materials	CNTs	PVA gels	1	2.06	[66]
Carbon materials	CB	TPU	1	1.80	[67]
Carbon materials	CNTs	SiO_2_/cellulose	1	1.25	[68]
Composite materials	CuO/CB	CB	1	1.65	[69]

**Table 2:** Evaporation performance of electrospun nanofiber materials based on different design strategies.

Design Strategy	Photothermal Material	Substrate	Characteristic	Solar Irradiance (kW m^−2^)	Evaporation Rate (kg m^−2^ h^−1^)	Ref.
Three-dimensional materials	CB	PET fibers	low cost and high efficiency	1	1.46	[12]
Design of single fibers	Carbon nanodots	PAN fibers	wide-spectrum light-trapping	1	1.73	[15]
Design of fiber membrane	CNTs	PAN fibers	good mechanical properties	1	1.28	[24]
Design of fiber membrane	CoOx/CB	Carbon fibers	ultra-high efficiency	1	3.23	[45]
Three-dimensional materials	CB	TPU fibers	high efficiency	1	1.80	[67]
Three-dimensional materials	CNTs	PI fibers	long-lasting desalination	1	2.08	[76]
Design of fiber membrane	Fe3O4	PAN fibers	desalination of hypersaline water	1	1.76	[90]
Design of fiber membrane	CNTs	PVDF fibers	high efficiency	1	1.37	[99]
Design of fiber membrane	Carbon spheres	PVDF-HFP fibers	Janus structure membrane	1	1.29	[106]
Design of single fibers	PDA/PPy	PI fibers	single fiber with multi-structure	1	~1.43	[107]
Design of single fibers	CB/CuO	PVDF-HFP fibers	broad-spectrum absorption	1	1.88	[108]
Design of fiber membrane	CNTs	PCL fibers	high efficiency	1	2.00	[113]
Design of fiber membrane	CB	PMMA/PAN fibers	Janus structure membrane	1	1.3	[114]
Three-dimensional materials	GO	PI fibers	three-dimensional structure	1	1.42	[118]
Design of fiber membrane	rGO	Cellulose acetate fibers	high efficiency	1	1.85	[132]

## Data Availability

Data are contained within the article.
